# Supplementary material for: Inferring variant-specific effective reproduction numbers from combined case and sequencing data
Source: eLife. 2026 Jul 9;14:RP104802. doi: 10.7554/eLife.104802 (PMC13349379; doi:10.7554/eLife.104802)
Supplement: Peer review history

# Editor report: eLife Assessment

**DOI:** 10.7554/eLife.104802.2.sa0

**Authors:** Eduardo L Franco

**Affiliations:** McGill University Canada

**Keywords:** Important, Solid

This study provides new **important** insights concerning pathogen variant-specific reproduction parameters from molecular sequencing and case finding. The methods for inferring which variants will likely emerge in subsequent epidemic cycles are **solid**. This article is of broad interest to infectious disease epidemiology researchers and mathematical modellers of the COVID-19 pandemic.

# Referee report: Reviewer #1 (Public review)

**DOI:** 10.7554/eLife.104802.2.sa1

In this manuscript, the authors describe a new method to more accurately estimate the fitness advantage of new SARS-CoV-2 variants when they emerge. This was a key public health question during the pandemic and drove a number of important policy choices during the latter half of the acute phase of the pandemic. They attempt to link fitness to expected wave size. The analyses are tested on data from 33 different US states for which the data were considered sufficient. The main novelty of the method is that it links the frequency of variants to the number of cases and thus estimates fitness in terms of the reproduction number.

The results with the new method appear to be more consistent estimates of fitness advantage over time, suggesting that the methods suggested are more accurate than the comparator methods.

Given that the paper presents a methodological advancement, the absence of a simulation study is a weakness. I am satisfied that the trends estimated via the different approaches suggest a useful advancement for a difficult problem. However, the work would have been considerably stronger if synthetic data had been used to illustrate without doubt how the revised method better captures underlying, pre-specified differences in fitness.

# Referee report: Reviewer #2 (Public review)

**DOI:** 10.7554/eLife.104802.2.sa2

Summary:

This study develops a joint epidemiological and population genetic model to infer variant-specific effective reproduction numbers Rt and growth advantages of SARS-CoV-2 variants using US case counts and sequence data (Jan 2021-Mar 2022). For this, they use the commonly used renewal equation framework, observation models (negative binomial with zero inflation and Dirichlet-multinomial likelihoods, both to account for overdispersion). For the parameterization of Rt, again, they used a classic cubic spline basis expansion. Additionally, they use Bayesian Inference, specifically SVI. I was reassured to see the sensitivity analysis on the generation time to check effects on Rt.

This is an incredibly robust study design. Integrating case and sequence data enables estimation of both absolute and relative variant fitness, overcoming limitations of frequency-only or case-only models. This reminds me of https://www.medrxiv.org/content/10.1101/2023.01.02.23284123v4.full

I also really appreciated the flexible and interpretable parameterization of the renewal equations with splines. But I may be biased since I really like splines!

The approach is justified, however, it has some big limitations. Specifically, there are some notable weaknesses, that I detail below.

(1) The model does not account for demographic stochasticity or transmission overdispersion (superspreading), which are known to affect SARS-CoV-2 dynamics and can bias Rt, especially in low incidence or early introduction phases.

(2) While the authors explore the sensitivity of generation time, the reliance on fixed generation time parameters (with some adjustments for Delta/Omicron) may still bias results

(3) There is no explicit adjustment for population immunity, which limits the ability to disentangle intrinsic variant fitness even though the model allows for inclusion of covariates - this to me is one of two major flaws in the study.

(4) The second major flaw in my opinion is that there is no hierarchical pooling across states - each state is modeled independently. A hierarchical Bayesian model could borrow strength across states, improving estimates for states with sparse data and enabling more robust inference of shared variant effects.

I would strongly recommend the following things in order of priority, where the first two points I consider critical.

(1) Implement a hierarchical model for variant growth advantages and Rt across states.

(2) Include time-varying covariates for vaccination rates, prior infection, and non-pharmaceutical interventions directly. This would help disentangle intrinsic variant transmissibility from changes in population susceptibility and behavior.

(3) Extend the renewal model to a stochastic or branching process framework that explicitly models overdispersed transmission.

(4) It would be good to allow for multiple seeding events per variant and per state. This can be informed by phylogeography in a minimum effort way and would improve the accuracy of Rt.

(5) By now, I don't think it will be a surprise that addressing sampling bias is standard, reweighting sequence data or comparing results with independent surveillance data to assess the impact of non-representative sequencing.
